# Characterization and Surface Study of Volcanic Ashes from Popocatépetl

**DOI:** 10.1021/acsomega.5c10174

**Published:** 2026-02-03

**Authors:** Nahomy Lazcano-González, Daniela Baéz-Prado, Stephany Natasha Arellano-Ahumada, María Alejandra Romero-Morán, Hugo Vazquez-Lima, Daniel Ramírez-Rosales, Yasmi Reyes-Ortega, Samuel Hernández-Anzaldo

**Affiliations:** † Centro de química, 3972 ICUAP. Benemérita Universidad Autónoma de Puebla. IC9 Laboratory 102. CU, 72570. Puebla, Puebla, Mexico; ‡ Escuela Superior de Física y Matemáticas, Depto. de Física, 27740 Instituto Politécnico Nacional, Edif. 9, U. P. Zacatenco, Col. San Pedro Zacatenco CDMX, 07738, Mexico; § Facultad de Ciencias Químicas, Benemérita Universidad Autónoma de Puebla. CU, 72570. Puebla, Puebla, Mexico

## Abstract

Puebla City, Mexico, experienced several volcanic ash storms that polluted the city and its surroundings. The spectroscopic characterization of the volcanic ashes is reported in more detail herein using IR, ESR, SEM, and powder diffraction X-ray. We report for the first time the use of volcanic ashes from Popocatépetl, a natural material, functioning as an adsorbent for removal of the cationic dye methylene blue from aqueous media. Batch adsorption experiments were conducted under varying initial dye concentrations and contact times to describe the adsorption behavior of the ashes, was obtained a SSA = 0.7245 m^2^/g. The equilibrium data were fitted to the Langmuir and Freundlich isotherm models. The Langmuir model exhibited a strong correlation (*R* ^2^ = 0.99351) that suggested monolayer adsorption on a homogeneous surface with a maximum adsorption capacity of *q*_max_ 3.46 mg/g. These findings support the notion that volcanic ash can act as a natural alternative adsorbent. The value of a pseudo-first-order kinetic constant *k* = 8.02 × 10^–3^ min^–1^ (*R* ^2^ = 0.99489) was obtained by batch equilibrium adsorption experiments when 40 mg/L of MB was used. This value, along with *q*_max_, is similar to activated carbon products from seeds. Remarkably though, adsorption by volcanic ashes did not require any additional treatment, unlike current carbon-based products.

## Graphical abstract

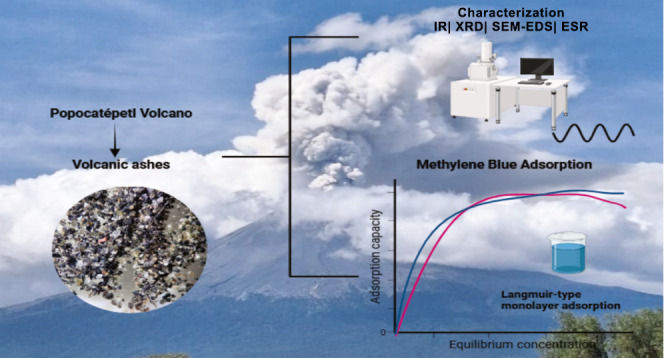

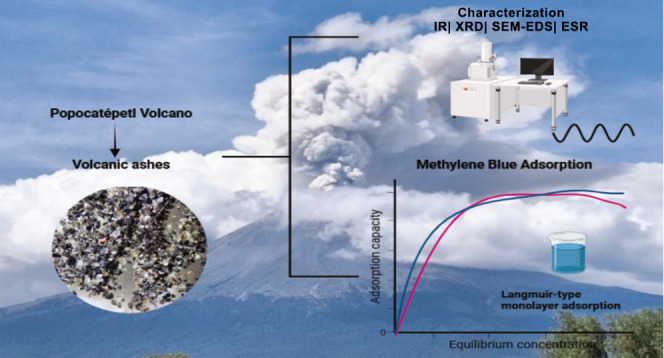

## Introduction

The active stratovolcano Popocatépetl is in central Mexico, 40 km from Puebla and 70 km from Mexico City. It is the second-highest volcano in Mexico, reaching 5452 m above sea level. The activity of this volcano has been resumed since 1960 to the present day.^1,2^ It plays an important role in climate regulation and, therefore, also in agricultural productivity; the use of volcanic ash as a natural fertilizer is feasible.^3–5^ In the periods of April–July and October–December of 2023, the activity of the Popocatépetl volcano was intensified in comparison to that reported in 2022.^6^ The reported geochemical composition indicates irregular shapes and sizes of volcanic ash, as well as high concentrations of SiO_2_, Al_2_O_3_, and Fe_2_O_3_.^5–7^ However, it also reports the presence of other elements such as Mg, Na, K, and Ca and Ti, according to several analyzed ashes, as well as the presence in smaller proportions of As, Ba, Br, S, and Sr.^5–8^ Additionally, volcanic ash from Popocatépetl reports a wide range of 3d metals, such as Cu, Fe, Ni, Co, and Ti and Pb as well,^2–9^ all of them in their cationic form and stabilized by the anion O^2–^. These silicates, aluminosilicates, and other oxides present in the volcanic ashes are like those used to adsorb methylene blue among others.^10–13^

Methylene blue (MB) is a versatile compound with numerous applications in different areas of study. This dye has significant chemical properties that facilitate its use in adsorption studies like optical and spectroscopic properties that are closely linked to its molecular structure characterized by a heterocyclic aromatic compound such as π–π interactions and proton transfer.^11,12^ The molecular structure carries a positive charge, enhancing its interaction with negatively charged adsorbents, Figure 1. Isothermal studies are the most frequent analyses to determine the efficiency and kinetics of MB adsorption; typically, Langmuir and Freundlich mathematical models are used to detail the adsorption process.^10,14^

**Figure 1:**
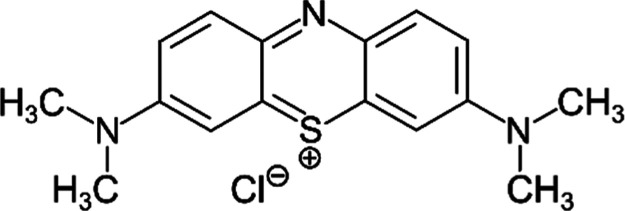
Molecular structure of methylene blue.

The adsorption efficiency and rates of MB on various adsorbents, like activated carbon (AC), agriculture waste, silica, sewage sludge,^14^ etc., have been extensively investigated. AC might be the most widely used adsorbent for MB’s removal according to its high adsorption capacity and fast removal time because of their high specific surface area.^15^ The origins of AC are various, from fruit peels or seeds to dry leaves and woods.

The AC activation and postactivation require high temperatures and several chemicals which are highly environmentally unfriendly and toxic processes.^14,15^ Moreover, the activation of AC has an average cost of USD 73, when natural precursors are used.^16^ Therefore, volcanic ash represents an innovative source, as it does not require pre- or postactivation treatment, which significantly reduces costs and environmental impact.

Notably, the reported adsorption properties are not exclusive to ashes from the studied volcano; ashes from other volcanoes contain basically the same materials but slightly different proportions. In this article, the physicochemical characteristics of volcanic ashes from the Popocatépetl explosions in May 2023 are discussed herein. The ashes properties matched minerals and other aggregates that have been previously used as natural adsorbents, making these volcanic ashes a material with potentially high use as a natural, less expensive, and effective adsorbent.

## Methodology

### Collection of Volcanic Ashes Samples

Eight samples of volcanic ashes were collected from different regions of Puebla, Mexico; at the very day of the eruption, the samples were collected in previously cleaned flat plastic containers, and a total of 4 kg was collected. Then, the ashes were ground and used in the adsorption experiments without further treatment, as indicated below: Samples A (April 24, 2023, Puebla city, Puebla), B (May 15, 2023, San Jerónimo Caleras, Puebla), C and D (May 17, 2023, San Jerónimo Caleras, Puebla), E (May 22, 2023, Puebla city, Puebla), F and G (May 22, 2023, Puebla city, Puebla), H (May 21, Huejotzingo, Puebla), and I and J (May 21, 2023, Puebla city, Puebla). From all the samples, A, D, G, H, and J were used after IR, X-ray diffraction, and SEM to prove their similarity and homogeneity in morphology, Figure S1.

### Physical Characterization of Volcanic Ashes

Infrared (IR): The samples of volcanic ashes in infrared FAR-IR and FTIR infrared spectra were measured in KBr pellets using a Nicolet Magna-IR 750 FT-IR spectrometer over the range of 4,000–400 cm^–1^. Far IR spectra were recorded with a (Perkin-Elmer 1600) spectrophotometer (400–75 cm^–1^). XRD: For powder angle-dispersive X-ray diffraction (ADXRD), a Panalytical-Empyrean diffractometer was used. The SEM micrographs were acquired in a JEOL JSM-7800F Scanning Electron Microscopy field emission microscope employing Secondary Electron Detection. An Oxford Instruments X-Max energy dispersive X-ray spectroscopy (EDS) system was used. The X-band ESR spectra of powdered samples were recorded in a Bruker ELEXSYS E500 II spectrometer at 300 and 77 K. Batch isotherms were acquired by using a Beckman DU 7500 UV/vis spectrophotometer at 664 nm in quartz cuvettes of 1 cm of length. For optical analysis, a Zeiss Stemi 508 microscope was used. Statistical analysis and nonlinear fitting were performed using Origin. Lab 8.1, Massachusetts, USA, 2024.

### Absorption Spectroscopy and UV–Vis of the Adsorbate: Methylene Blue

Methylene blue (MB) supplied by Sigma-Aldrich was used as the adsorbate and was not purified prior to use. Distilled water was employed for preparing all of the solutions and reagents. MB has a molecular weight of 373.9 g/mol, which corresponds to MB hydrochloride with three groups of water. The concentration of MB in the solutions before and after the adsorption was determined by UV–vis. The calibration curves were always reproducible in the concentration range used.

### Batch Equilibrium and Kinetic Studies

Adsorption isotherms were acquired in a set of 15 Falcon tubes (15 mL), where solutions of dye with different initial concentrations (10 to 100 mg/L) were placed in the tubes. An amount of 0.05 g of volcanic ashes was added to dye solutions, and each sample was kept at 22 °C for 24 h to reach equilibrium. A similar procedure was performed in a group with no volcanic ashes as the control for the experiments. All experiments were conducted with five replicates for statistical purposes, Figures S2–S5.

The amount of adsorption of MB at equilibrium, *q*_e_ (mg/g), was calculated by eq 1:

1 $$q_{e}=\frac{\left(C_{0}-C_{e}\right)V}{W}$$

where *C*_0_ and *C*_e_ (mg/L) are the liquid-phase concentrations of the dye initially and at equilibrium, respectively, *V* is the volume of the solution (L), and *W* is the mass of the dry adsorbent used (g).

Kinetic experiments were identical to those of the equilibrium tests. The samples were taken at different time points, and the concentrations of MB were similarly measured. The amount of adsorption at time, *t*, *q*_*t*_ (mg/g), was calculated by eq 2

2 $$q_{t}=\frac{\left(C_{0}-C_{t}\right)V}{W}$$

where *C*_0_ and *C*_*t*_ (mg/L) are the liquid-phase concentrations of MB dye initially and at any time *t*, respectively, *V* is the volume of the solution (L), and *W* is the mass of dry adsorbent used (g).

## Results and Discussion

### Characterization of Volcanic Ashes

#### Optical Morphology and IR

The use of optical microscopes helped us to identify several changes in the morphology and sizes of the ashes, Figure 2. However, the medium and far IR spectra show that the composition is the same from different places where the samples were taken, indicating that the dominant chemical bonding environments are similar across all sampling sites. The bands at approximately 1100 cm^–1^ correspond to Si–O asymmetric stretching modes typical of silicate structures, while the features between 470 and 580 cm^–1^ are associated with M–O bending vibrations (M = Fe, Cu, Zn) commonly found in metal-oxide environments. The broad absorption near 3442 cm^–1^ arises from O–H stretching of adsorbed water or hydroxyl groups.^17–23^

**Figure 2:**
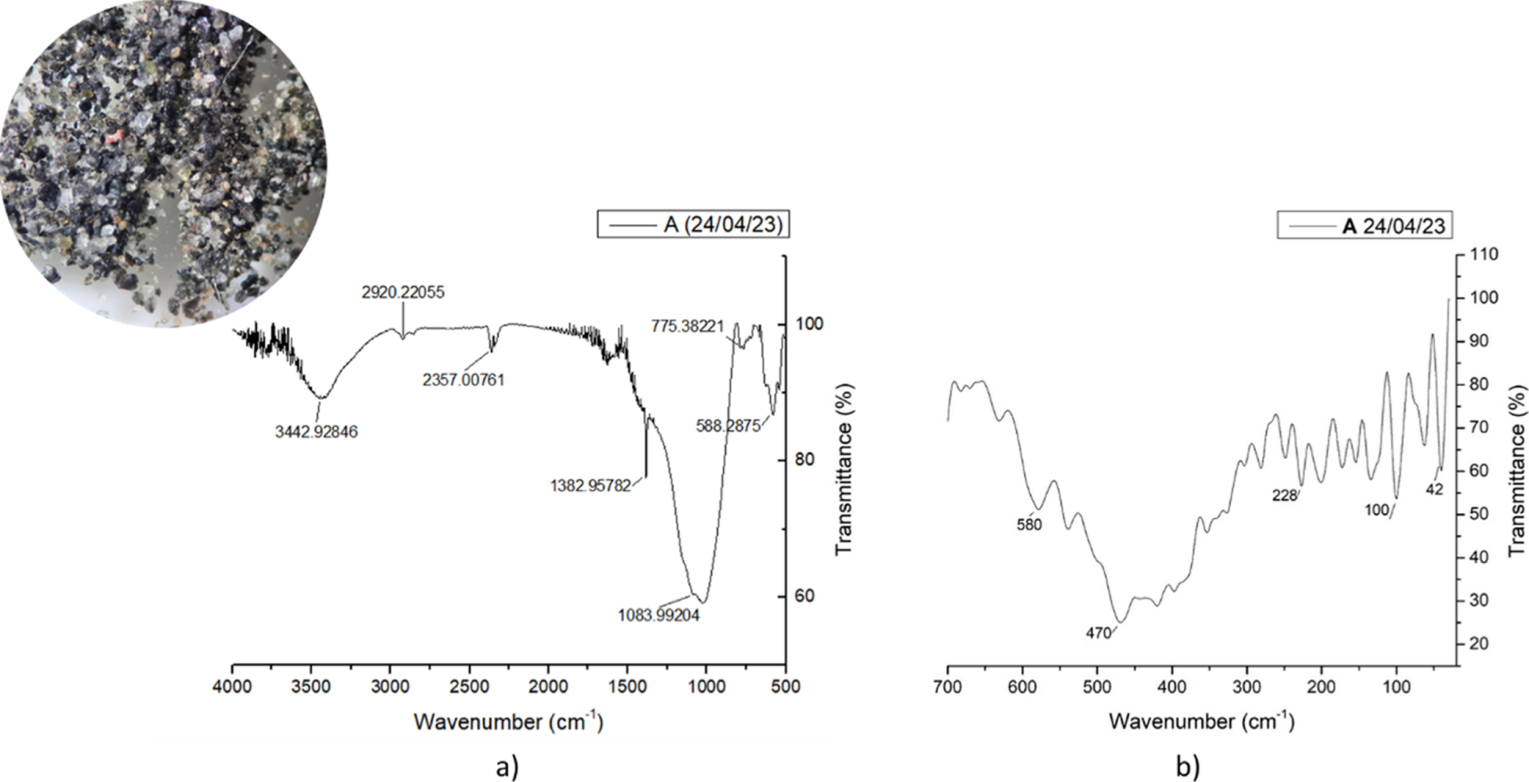
Sample A, volcanic ashes from Puebla, Puebla. April 24, 2023. (a) FTIR spectra and (b) FAR-IR spectra. Left corner inset, volcanic ashes under a microscope.

It is important to note that IR spectroscopy detects vibrational transitions of chemical bonds rather than unambiguously identifying crystalline phases such as SiO_2_, Fe_2_O_3_, or CuO. Although these vibrational modes are consistent with the structural motifs present in those phases, their assignment to specific minerals is not based solely on IR values but on the overall spectroscopic analysis. This interpretation aligns with previous reports showing that volcanic ash exhibits similar silicate and metal-oxide bonding frameworks.

The different aspect of the ashes may be due to the erosion of the solid particles along their path before deposition on the soil.^17^ According to different scientific articles, many types of volcanic ashes have slightly different content, and the variation mainly comes in metallic composition percentage.^18,19^ It is important to highlight that the composition is mainly of monofunctional ionic substances with very strong intermolecular attraction, and more metallic alloys are present in the volcanic ashes as we will discuss in the following sections. However, the low net dipole moment that they generate decreases their vibrational transition permittivity agreeing to the infrared selection rules.^23^ This phenomenon does not allow for the certain assignment of the bands for those alloys.

#### X-ray Powder Diffraction (XRD)

The XRD diffractogram of volcanic ashes shows crystalline phases of several ionic metallic traces typical of volcanic ash.^8,9^ The XRD patterns of all samples show the existence of slight amorphous phase suggested by the signal broad with a 2θ from 5 to 10, which is typically assigned to no crystalline material or amorphous solids.^20–23^ The result might arise from the relatively rapid cooling of volcanic lavas plus the changes from the airtime which also have been studied previously.^20^ Figure 3 shows the XRD of the powder samples. The composition by this analysis is quartz (SiO_2_), (Si,Al)_4_O_8_, and hematite (Fe_2_O_3_) as major constituents while diopside (MgCaSi_2_O_6_) and albite (NaAlSi_3_O_8_) as minor components. The ionic inorganic solids are popular components of efficient adsorbents for their interaction with dyes like MB, since the anionic part could interact with the positive charge of MB and the cation would be stabilized by the chloride from MB as well.

**Figure 3:**
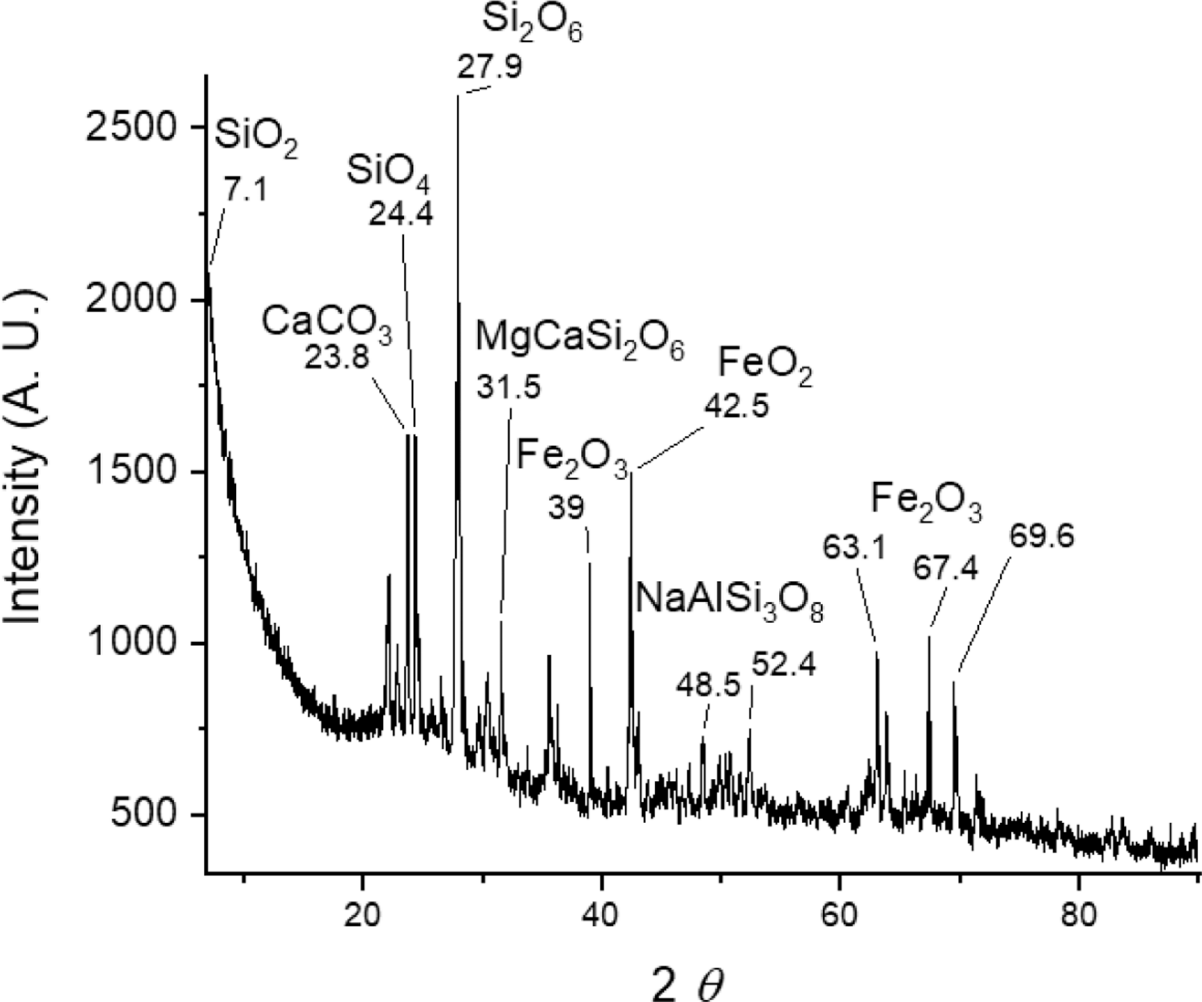
XRD diffractogram of volcanic ashes from Popocatépetl.

### ESR Spectroscopy

The ESR spectra determine the oxidation states and the magnetic environment of the metallic ions present in the solids coming from the volcanic ashes. The technique can analyze species with unpaired electrons, and its high precision was crucial to unveiling the composition of metals and their oxidation state. First, the singlet rhombic distorted signal typically occurs in minerals and glasses such as volcanic ashes, Figure 4. No magnetic exchange interactions are contributing to the broadness of the signal; this may be due to the dilution coming from the diamagnetic metals like the electron close-shell cations: Na­(I), Ca­(II), Mg­(II), and Si­(IV) are not seen in the ESR spectra.

**Figure 4:**
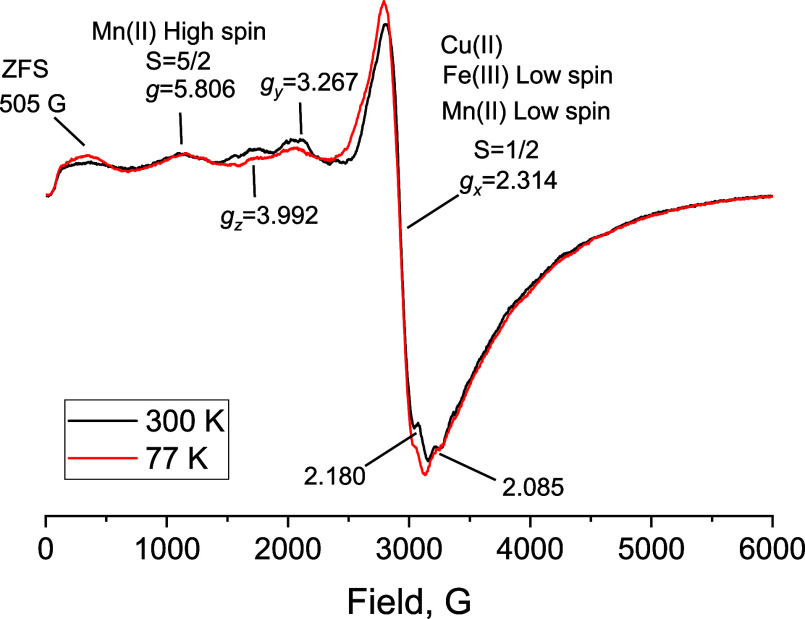
Volcanic ashes X-band ESR spectra at 300 and 77 K.

The spectra show the presence of several paramagnetic species with low spin (LS) and high spin (HS) configurations, Figure 4. The *g* values reported are typical for Cu­(II), low spin Mn­(II), and low spin Fe­(III), all of them with *S* = 1/2 and *g* = 2.314, as well as high spin Fe­(III) *S* = 5/2 with *g* = 5.806.^21,22^ The spectrum has a rhombic line shape; this is due to the high anisotropic behavior of the metallic ions in the volcanic ashes affecting the electronic momentum of the uncoupled electrons. This anisotropy leads to the splitting of electronic magnetic states without an applied magnetic field known as Kramer’s doublets and generating the Zero Field Splitting (ZFS) which is seen at 505 G for this case.^23^ The other *g* values refer to the components of the tensor *g* from the *S* = 1/2 species; these components are *g*_*x*_ = 2.314, *g*_*y*_ = 3.267, and *g*_*z*_ = 3.992. The spectrum at 77 K shows a slight increase of the spectral area due to the higher electronic spin population without the thermal effect.^23^

Giles describes that paramagnetic and diamagnetic inorganic cations, like those present in the volcanic ashes, are related to the ion-exchange affinities of inorganic ions on resins or dyes and their exchange isotherms, with isotherm types of S, C, or L class, L in our work, as we will discuss in the adsorption section.

#### Scanning Electron Microscopy (SEM) and Energy-Dispersive X-ray Spectroscopy (EDS)

To fully characterize the volcanic ashes, its shape and morphology, SEM-EDS analysis was conducted. To confirm the presence of transitional metals, multiple regions of the ashes were analyzed, revealing consistent patterns, Figures 5 and S7.

**Figure 5:**
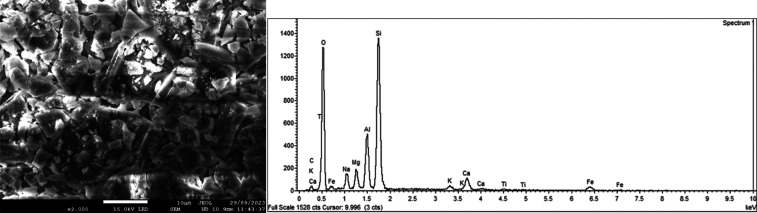
SEM-EDS of volcanic ashes. Left: Micrograph. Right: Energy binding vs absorption from the EDS analysis.

Additionally, the images of the volcanic ashes demonstrate flatness of the material could help in horizontal adsorption that in some cases is studied by isotherms type L2 according to Giles et al.^24^ and the shape and sizes are diverse but no bigger than 10 μm.

### Inductively Coupled Plasma-Mass Spectrometry

To confirm the presence of transition metals, multiple samples of volcanic ashes were analyzed, revealing consistent results along with the other techniques presented previously and those of other authors. Nickel was confirmed by inductively coupled plasma-MS in the treated groups, and it is present abundantly in the volcanic ashes, as shown in Figure 5. It is observed that the other metals that are also present in the SEM-EDS and ESR except for Zn­(II) since this oxidation state of zinc has not unpaired electrons.^23^ The detection of nickel was an unexpected finding, as it was not detected by other techniques, Figure 6, since the Ni­(II) presents an *S* = 1, and it does not display a signal using ESR, for example.

**Figure 6:**
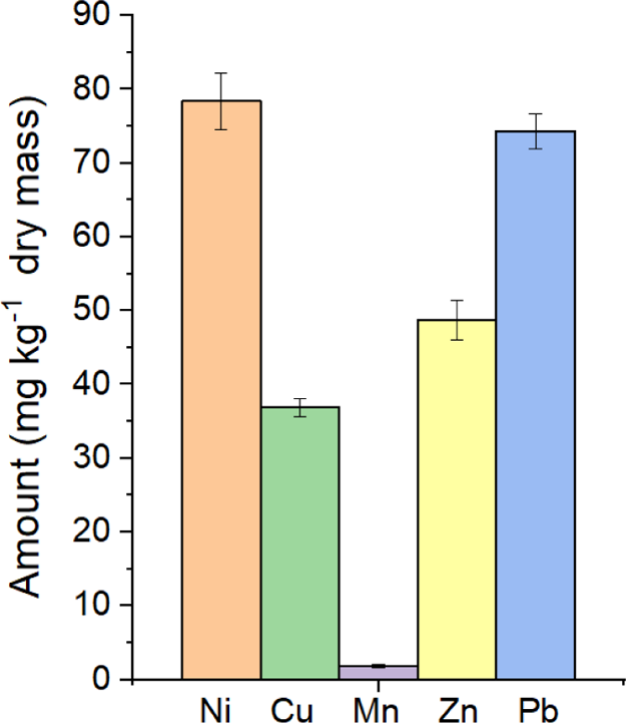
Inductively coupled plasma-mass spectrometry analysis from volcanic ashes.

### Adsorption Isotherms

Since spectroscopic and physical analyses show similarity to adsorbent materials, adsorption experiments were performed using adsorption isotherms. Isotherm models are an effective way to anticipate adsorption behavior and investigate the interactions of adsorbents and pollutants in an equilibrium medium. An adsorption isotherm indicates how the adsorption molecules distribute between the liquid phase and the solid phase when the adsorption process reaches equilibrium. The analysis of the isotherm data by mathematical fitting to different isotherms may be used to propose alternative materials for removing pollutant dyes, for example.^15^ The concentration of MB dye adsorbed (*q*_e_) is plotted versus the equilibrium concentration (*C*_e_) in Figure 7. The equilibrium adsorption density, *q*_e_ increased with the increase in dye concentration, Figures S6 and S7. The Langmuir and Freundlich models, eqs 3 and 4, are the most frequently employed models. In this work, the two models were used to describe the relationship between the amount of dye adsorbed and its equilibrium concentration.

3 $$q_{e}=\frac{q_{\max}\cdot k\cdot C_{e}}{\left(1+k\cdot C_{e}\right)}$$

4 $$q_{e}=k\cdot C_{e}^{1/n}$$

where *q*_max_ denotes the maximum adsorption ability, *C*_e_ is the concentration at the equilibrium state, and *q*_e_ is the equilibrium adsorbent concentration. The Langmuir constant *k* corresponds to the strength of molecule adsorption on the adsorbent surface for eq 3, and for this case, *k* = 1.048 L/g. The higher *R* ^2^ suggests that the Langmuir model provides a better fit to our adsorption process, Figure 7. The fitting for the Langmuir model justifies the canonic assumption that the sorption takes place at specific homogeneous sites within the volcanic ashes, and it was performed over the *C*_e_ vs *q*_e_ plot using eq 3.

**Figure 7:**
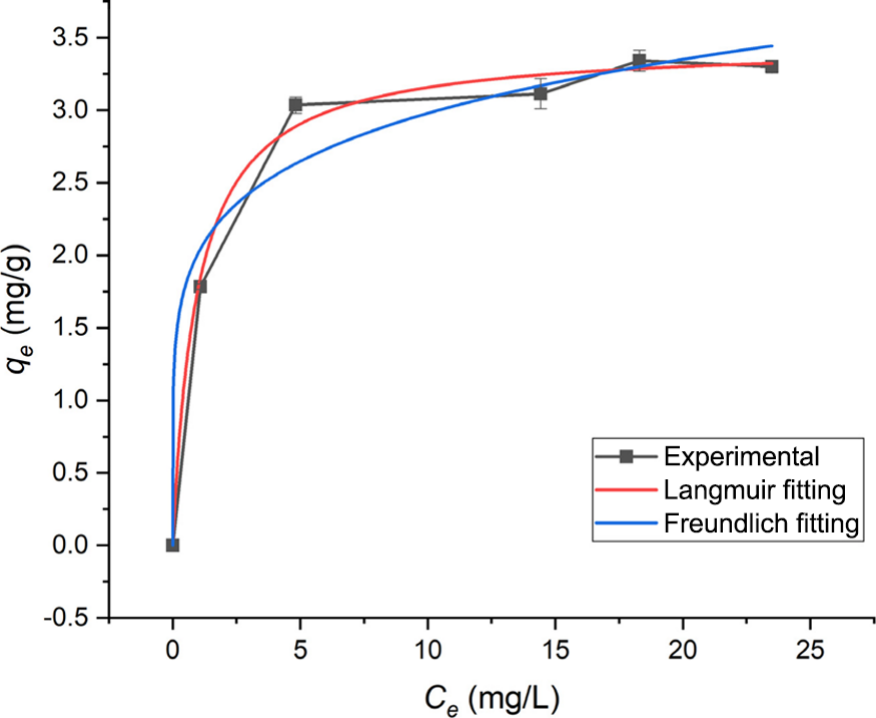
Adsorption isotherm. Langmuir (*R* ^2^ = 0.99351) and Freundlich (*R* ^2^ = 0.96349).

According to refs 26 and 27 , the maximum adsorption capacity (*q*_max_) can be related to adsorption to calculate the total surface area and, therefore, the specific surface area, eq 5:

5 $$SSA=q_{\max}\cdot W\cdot \frac{N}{m}$$

where *W* is the effective cross-sectional area of one adsorbate molecule, *N* is the Avogadro constant, and *m* is the mass of the volcanic ashes used. For the analyzed material, the SSA calculated by this method is SSA = 0.7245 m^2^/g, which is in the range of materials with no treatment and other silica.^25^

According to Giles, the experimental isotherm behaves as an L2 adsorption curve, allowing us to establish a direct relationship between the structure of volcanic ash and its adsorption mechanism. Popocatépetl ash has highly heterogeneous surfaces, as well as fragmented morphologies derived from rapid eruptive processes. This combination generates a distribution of adsorbent sites with variable accessibility. Under these structural conditions, the L2 isotherm reflects that the adsorption of methylene blue (MB) begins with the occupation of the most easily accessible sites and continues toward progressively less available regions, which increases the difficulty of incorporating new molecules as the pores and cavities become saturated.^24^

The presumably flat orientation of MB on these surfaces is consistent with the laminar geometry of the dye and the presence of siliceous and ferric areas capable of establishing π–surface interactions and weak electrostatic forces. Furthermore, the limited competition between water and MB suggests that the surface groups of the ashes favor direct adsorption of the dye, probably through surface interactions dominated by textural heterogeneity rather than strong displacement processes.^24–26^

This interpretation is consistent with the specific surface area values obtained by N_2_ adsorption (SSA = 1,225 m^2^ g^–1^) in previous reports,^7^ which show a porous texture sufficient to accommodate larger organic molecules. These properties, combined with the irregular morphology of volcanic particles, reinforce the concept that ash acts as an effective adsorbent for dyes, whose mechanism is based on progressive adsorption, oriented in a flat manner and dependent on the structural heterogeneity of the material.^7,24^

The kinetics for the process presented by the isotherm herein are reported to fit pseudo-first-order kinetics, Figure 8. The best fitting for the Langmuir isotherm adsorption by the volcanic ashes is calculated by eq 6:

**Figure 8:**
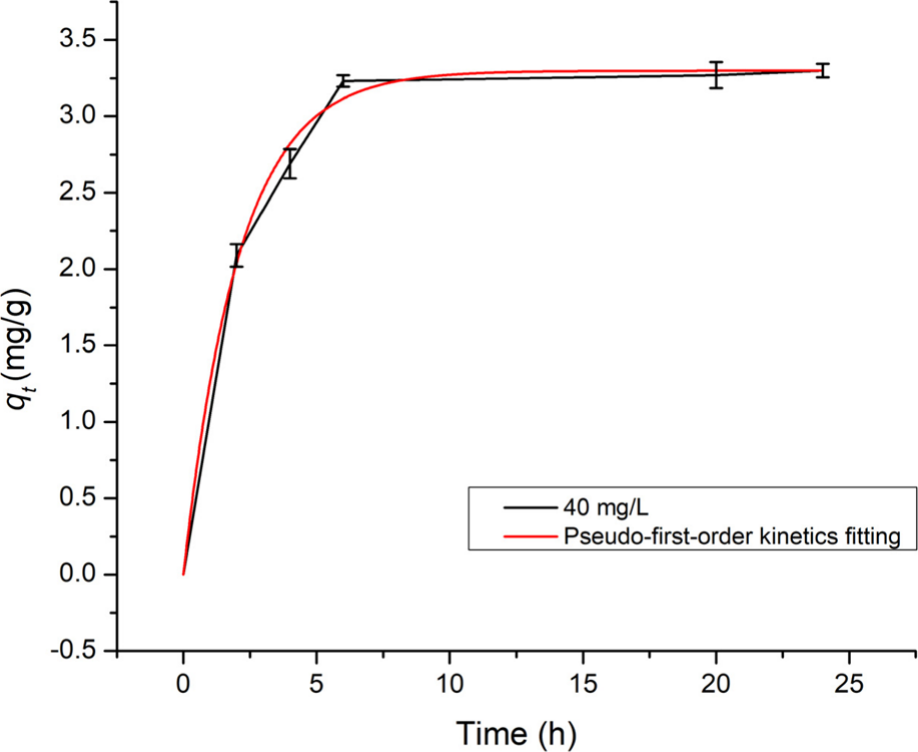
Pseudo-first-order kinetics fitting and experimental 40 mg/L MB adsorbance over 24 h.

### Pseudo-First-Order Kinetics

6 $$q_{t}=q_{e}\left(1-e^{-kt}\right)$$

where *q*_e_ and *q*_*t*_ are the sorption capacity at equilibrium and at any time *t*, respectively, and *k* is the pseudo-first-order rate constant, min^–1^.

The kinetic parameters of MB adsorption by volcanic ashes and its comparison with those of selected materials are shown in Table 1. The comparison shows that volcanic ash is competitive with natural materials and some artificial adsorbents; however, the most notable characteristic of ash is its good adsorption capacity without requiring additional treatment, as clean volcanic ash could facilitate the adsorption of dyes for their removal, for example.

**Table 1:** Comparative *q*_max_ for Different Adsorbents^28–31^

adsorbent	*q*_max_ mg/g	reaction order	reference
volcanic ashes (without treatment)	3.4	pseudo-first order	in this study
activated carbon synthesis from pepper stem	178.42	pseudo-second order	(Dolas, 2023)
activated carbon synthesis from coffee husk	6.82	pseudo-second order	(Ayalew and Aragaw, 2020)
activated carbon synthesis from tamarind seed	1.24	pseudo-second order	(Ishak et al., 2021)
natural magnetic sand	1.01	pseudo-first order	(Ozer, 2020)

An important consideration for practical applications is potential metal leaching. Previous characterization of Popocatépetl ash by Santamaría-Juárez et al.^32^ demonstrated minimal metal leaching under aqueous conditions, with concentrations below detection limits for most elements. This low leaching is attributed to stable crystalline phases identified in our XRD analysis, which are thermodynamically stable in neutral pH aqueous environments.

## Conclusion

Volcanic ashes from Popocatépetl were characterized by spectroscopic techniques that demonstrated that the material contained Fe­(III), Mn­(II), Cu­(II), Zn­(II), Ni­(II), and Mg­(II). The morphology and size of the ashes were also described. Their composition compares with those of materials that have been successfully utilized as an adsorbent for the quantitative removal of MB from aqueous solution. Equilibrium adsorption was achieved within 24 h. The isothermal adsorption data fits in the Langmuir model with a L2 shape curve, suggesting that ashes not only favor adsorption but also position them as efficient materials for capturing organic dyes in monolayer coverage of dye molecules in the outer surface. The kinetics of MB adsorption into volcanic ashes followed the pseudo-first-order model with a *k* = 0.00802 min^–1^. There are several studies of volcanic ash in other areas; however, this is the first time that volcanic ash from the Popocatépetl volcano has been used as a methylene blue absorbent without any pre/post-treatment activation. Therefore, volcanic ashes could be used as a low-cost alternative adsorbent in processes such as chemical purification or removal of dye from wastewater.

## Supplementary Material



## Notes

Infrared spectra of volcanic ashes (Figure S1); pseudo-first-order kinetics fitting and experimental 35 mg/L MB absorbance over 24 h (Figure S2); pseudo-first-order kinetics fitting and experimental 30 mg/L MB adsorbance over 24 h (Figure S3); pseudo-first-order kinetics fitting and experimental 20 mg/L MB adsorbance over 24 h (Figure S4); pseudo-first-order kinetics fitting and experimental 10 mg/L MB absorbance over 24 h (Figure S5); and treatments of volcanic ashes with different concentrations of MB after 24 h (Figure S6) (PDF)
