# Supplementary material for: Characterization and Surface Study of Volcanic Ashes from Popocatépetl
Source: ACS Omega. 2026 Feb 3;11(6):9704–10. doi: 10.1021/acsomega.5c10174 (PMC12917834; doi:10.1021/acsomega.5c10174)
Supplement: Supplementary file 1 [file ao5c10174_si_001.pdf]

SUPPORTING INFORMATION

Characterization and surface study of volcanic ashes from Popocatépetl

Nahomy Lazcano-González [a], Daniela Baéz-Prado [a], Stephany Natasha Arellano-Ahumada [b], María Alejandra Romero-Morán [a], Hugo Vazquez-Lima [c], Daniel Ramírez-Rosales [b], Yasmi Reyes-Ortega [a] * and Samuel Hernández-Anzaldo [a] *

[a], Centro de química, ICUAP. Benemérita Universidad Autónoma de Puebla. IC9 Laboratory 102. CU, 72570. Puebla, Pue. Mexico.
[b], Instituto Politécnico Nacional, Escuela Superior de Física y Matemáticas, Depto. de Física, Edif. 9, U. P. Zacatenco, Col. San Pedro Zacatenco, CDMX, 07738, México
[c], Facultad de Ciencias Químicas, Benemérita Universidad Autónoma de Puebla. CU, 72570. Puebla, Pue. Mexico.

Corresponding author contact: samuel.hernandezan@correo.buap.mx, yasmi.reyes@correo.buap.mx

Key words: Popocatepetl volcano, metal composition, isotherm, methylene blue, kinetics adsorption.

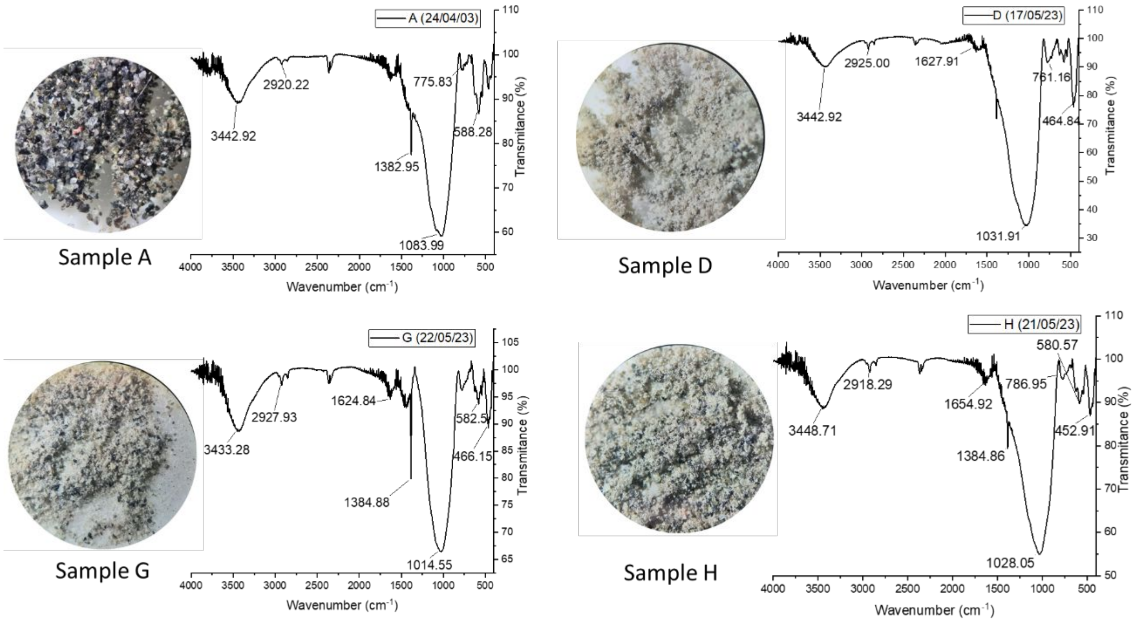

Figure S1. Infrared spectra of volcanic ashes. A) April 24, 2023 from Puebla D) May 17, 2023 from San Jerónimo G) May 22, 2023 from Puebla H) May 21, 2023 from Huejotzingo.

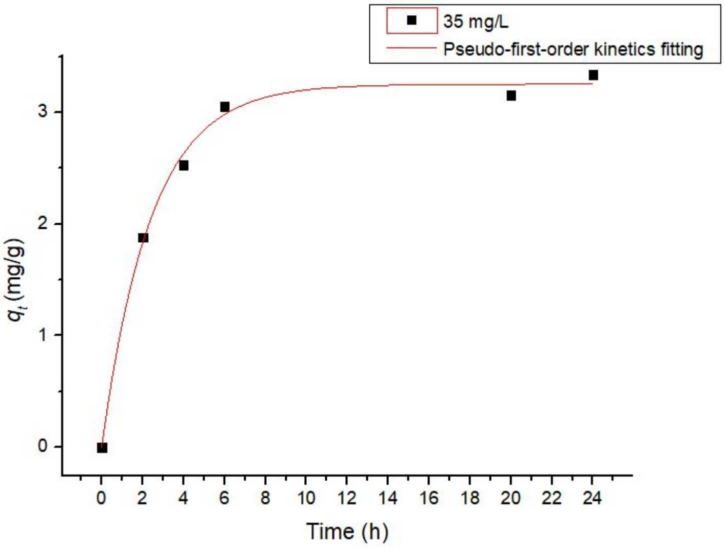

Figure S2. Pseudo-first-order kinetics fitting and experimental 35 mg/L methylene blue absorbance over 24 hours.

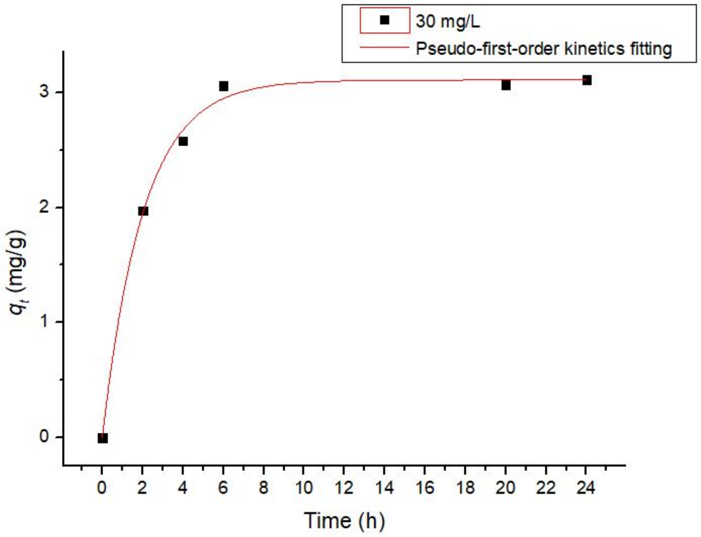

Figure S3. Pseudo-first-order kinetics fitting and experimental 30 mg/L methylene blue absorbance over 24 hours.

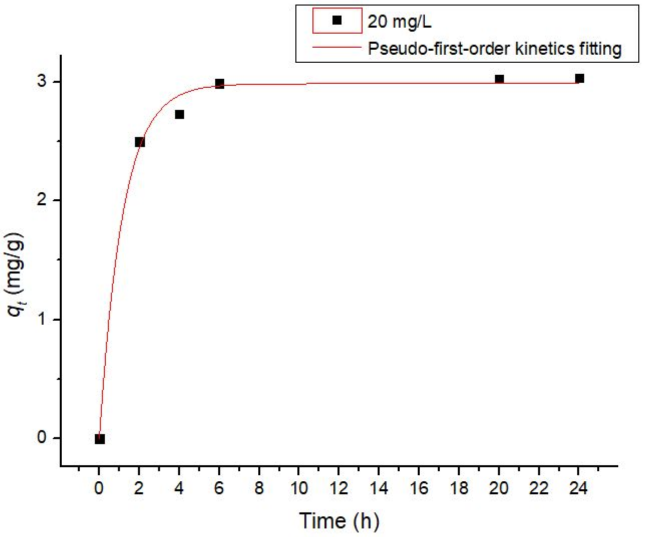

Figure S4. Pseudo-first-order kinetics fitting and experimental 20 mg/L methylene blue adsorbance over 24 hours.

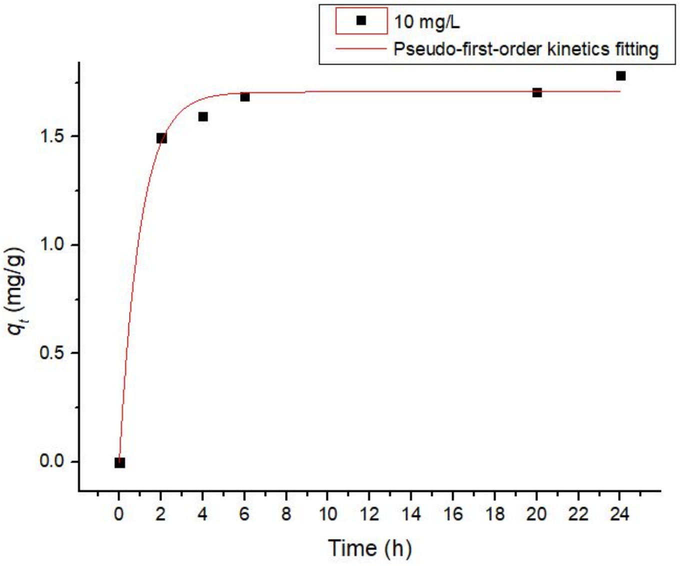

Figure S5. Pseudo-first-order kinetics fitting and experimental 10 mg/L methylene blue absorbance over 24 hours.

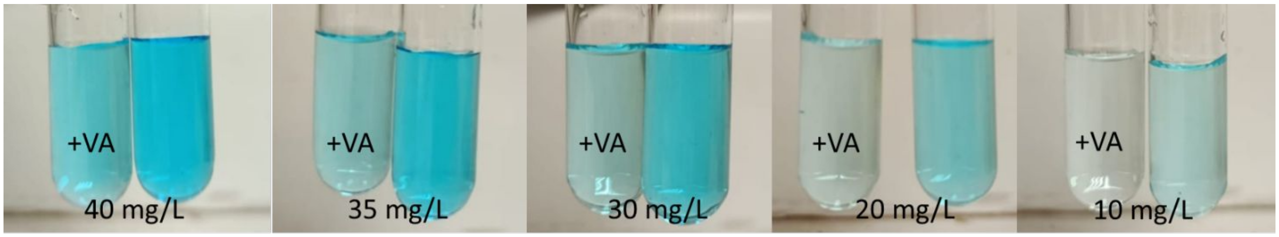

Figure S6. Treatments of volcanic ashes with different concentrations of MB after 24 hours.

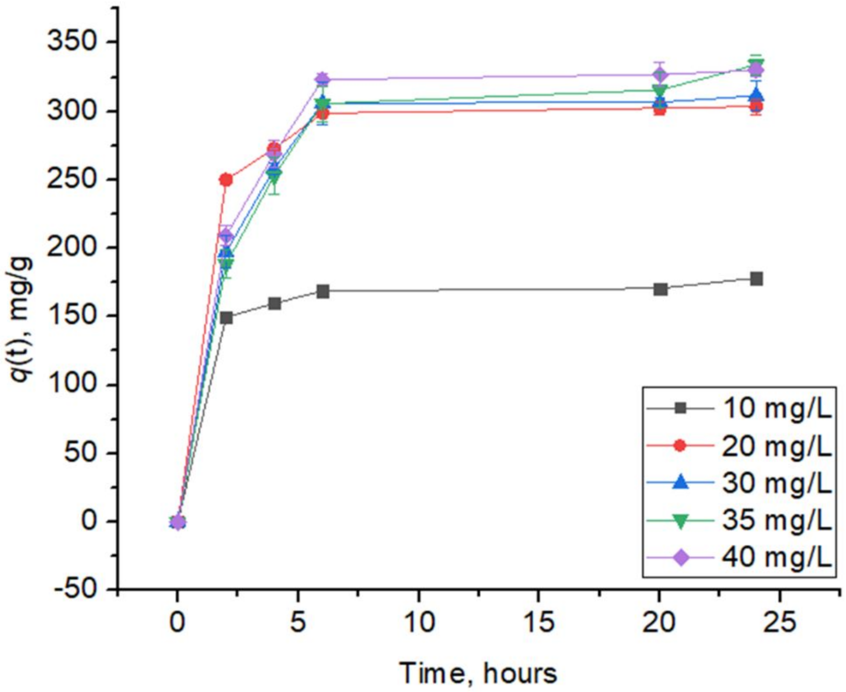

Figure S7. Equilibrium adsorption isotherm of several concentrations of methylene blue for 25h.
